# Population‐level diagnostic and radiation treatment trends of low‐risk prostate cancer

**DOI:** 10.1002/cncr.70603

**Published:** 2026-10-26

**Authors:** Steven M. Monda, Varshini Odayar, Maggie Yin, Rachel Tucker Gonzalez, Salvador Jaime‐Casas, Karolyn M. Hopfensperger, Danielle Kendrick, Melissa A. Mietzel, Mazen Mislmani, Mark Zaki, Patrick W. McLaughlin, Todd M. Morgan, William C. Jackson, Matthew J. Schipper, David K. Heimburger, Alex K. Bryant, Lori J. Pierce, Tudor Borza, Robert T. Dess

**Affiliations:** ^1^ Department of Urology University of Michigan Ann Arbor Michigan USA; ^2^ Department of Radiation Oncology University of Michigan Ann Arbor Michigan USA; ^3^ VA Ann Arbor Healthcare System Ann Arbor Michigan USA; ^4^ West Michigan Cancer Center Kalamazoo Michigan USA; ^5^ Covenant HealthCare Saginaw Michigan USA; ^6^ Department of Radiation Oncology Assarian Cancer Center Henry Ford Providence Hospital Novi Michigan USA; ^7^ Department of Biostatistics University of Michigan Ann Arbor Michigan USA; ^8^ Munson Healthcare Traverse City Michigan USA

**Keywords:** active surveillance, early detection of cancer, low‐risk prostate cancer, overtreatment, population surveillance, prostate‐specific antigen, PSA screening, quality improvement, radiation, radiotherapy

## Abstract

**Background:**

Overtreatment of low‐risk prostate cancer (PC) undermines screening efforts. Active surveillance is preferred for low‐risk PC, but declining treatment may reflect diagnostic or treatment‐selection changes. We assessed diagnostic risk‐group trends among localized PC and low‐risk trends among radiation‐treated patients since 2010.

**Methods:**

We analyzed localized PC in Surveillance, Epidemiology, and End Results Program (SEER; 2010–2023); Veterans Affairs (VA; 2010–2024); and Michigan Radiation Oncology Quality Consortium (MROQC; 2021–2025). Low‐risk PC was defined as grade group 1, prostate‐specific antigen <10 ng/mL, and clinical stage ≤T2a. Diagnosis‐based analyses described risk‐group trends among all localized PC diagnoses in SEER and VA and treatment among low‐risk patients. The primary outcome was the proportion of radiation‐treated patients classified as low‐risk, evaluated using logistic regression.

**Results:**

Among localized PC, low‐risk disease at diagnosis declined from 28.8% to 24.0% in SEER and from 32.3% to 21.9% in VA. Among diagnosed low‐risk patients, receipt of neither radiation nor prostatectomy increased from 33.6% to 83.4% in SEER and from 51.6% to 96.3% in VA. Among 207,196 patients treated with radiation for localized PC (170,213 SEER; 34,635 VA; and 2348 MROQC), the proportion with low‐risk disease declined from 33.7% to 6.5% in SEER, from 22.9% to 2.0% in VA, and remained ≤4.2% in MROQC. Declines persisted after adjustment.

**Conclusion:**

Since 2010, low‐risk PC has become less common at diagnosis, whereas diagnosed low‐risk patients increasingly avoid treatment. Radiation for low‐risk PC has markedly declined, paralleling surgical trends and reflecting a nationwide shift toward active surveillance.

## Abstract 2

Since 2010, low‐risk prostate cancer has become less common at diagnosis and is increasingly managed without definitive treatment. Among 207,196 radiation‐treated patients, low‐risk disease declined sharply, supporting a contemporary shift toward surveillance that counters overtreatment concerns historically cited against screening.

## INTRODUCTION

Prostate cancer (PC) is the most common malignant neoplasm and the second‐leading cause of cancer‐related death among men in the United States. During the 2010s, recommendations supporting prostate‐specific antigen (PSA)‐based PC screening wavered amid growing concerns of overdiagnosis and overtreatment of indolent disease.^1^, ^2^

Concurrently, PC management shifted with the establishment of guideline‐supported active surveillance for low‐risk PC.^3^ The extent to which this shift in management has translated into reduced treatment among the patients least likely to benefit is less well defined.

Our group has previously documented significant declines in the surgical overtreatment of PC.^4^ However, observed treatment trends may reflect both changes in disease presentation and changes in treatment selection among patients with low‐risk disease. Approximately one‐third of patients with localized PC are initially managed with radiation therapy, and it is unclear whether selection of patients for radiation has similarly evolved to exclude low‐risk disease.^5^ Men foregoing surgery may instead receive radiation, raising concerns that overtreatment with surgery is being shifted rather than reduced.

To contextualize contemporary treatment patterns, we first evaluated diagnostic risk‐group trends among all patients diagnosed with localized PC in two U.S. cohorts. We then examined whether the proportion with low‐risk disease among radiation‐treated patients across three U.S. cohorts has declined over time.

## MATERIALS AND METHODS

We analyzed three cohorts in parallel: the Surveillance, Epidemiology, and End Results Program (SEER) 17 Registries from January 1, 2010, to December 31, 2023; the Veterans Affairs (VA) health system from January 1, 2010, to December 31, 2024; and the Michigan Radiation Oncology Quality Consortium (MROQC) from statewide inception on January 4, 2021, to August 4, 2025. We performed two complementary analyses. First, using SEER and VA data, we evaluated annual diagnostic risk‐group trends among all patients with localized PC, irrespective of treatment. Second, using SEER, VA, and MROQC data, we evaluated the proportion of radiation‐treated patients with intact, localized PC who had low‐risk disease over time. Low‐risk PC was defined as grade group 1 on biopsy, PSA <10 ng/mL, and clinical stage T1‐T2a, consistent with National Comprehensive Cancer Network, American Urological Association, and European Association of Urology definitions; active surveillance is now the preferred management strategy for this group across guidelines.^6^, ^7^, ^8^

The VA cohort included medical record data from the Veterans Health Administration Corporate Data Warehouse.^9^ MROQC is a Michigan statewide radiation quality improvement collaborative consisting of 27 facilities. Michigan/Detroit is not included in SEER 17 and VA patients are incompletely captured within SEER because of data‐sharing restrictions. Given this, we treated SEER, VA, and MROQC cohorts as distinct, largely nonoverlapping populations, and performed all analyses strictly in parallel.^10^, ^11^

For the diagnosis‐based analysis, we identified patients in SEER and the VA diagnosed with localized PC (N0 M0) with known PSA and clinical Gleason grade between 2010 and 2023 in SEER and between 2010 and 2024 in the VA. We described annual risk‐group distributions among all localized diagnoses and, among low‐risk patients, primary treatment within 1 year of diagnosis categorized as radiation, prostatectomy, or neither. In SEER, clinical T‐stage was not reliably available for surgical patients in certain diagnosis years; T‐stage was therefore omitted from SEER risk‐group assignment in this diagnosis‐based analysis only.

For the radiation‐treatment analysis, inclusion criteria were applied consistently across the three cohorts. We included only patients captured within each cohort receiving radiation as initial treatment for localized PC, excluding those with metastatic or nodal disease (M+ or N+), and required a known PSA value and Gleason score.

Among radiation‐treated patients, low‐risk PC status was modeled as a binary outcome (low‐risk vs non–low‐risk) using logistic regression. Treatment year (continuous, scaled per 5 years), age (continuous, scaled per 5 years), and race (White [reference], Black, Other, Unknown/Missing) were included as fixed effects. To account for variation within sites, a random intercept was included for SEER‐17 registry in the SEER model and for treating facility in the VA and MROQC models. We also compared the proportion of low‐risk PC between SEER, VA, and MROQC in the years shared between all cohorts (2021–2023) using pairwise two‐proportion z‐tests.

SEER captures initial PC diagnosis but not subsequent diagnoses (e.g. upgrading on later biopsy). To minimize misclassification of patients initially managed with active surveillance who later underwent definitive treatment because of undocumented upgrading, we restricted the radiation‐treatment analysis to patients treated within 12 months of diagnosis. This timeframe corresponds to typical active surveillance protocols in which confirmatory biopsy and risk reassessment are performed within the first year after diagnosis. This restriction excluded 10,574 patients in SEER (6.4%), 1,61 in the VA (4.3%), and 212 in MROQC (8.3%) from the otherwise‐eligible cohort.

Within the VA and MROQC, where facility‐level data were available, the proportion of radiation‐treated patients with low‐risk cancer was reported at each facility. This was assessed for 116 facilities in the VA and 26 facilities in Michigan.

SEER and VA analyses were performed using R version 4.5.1 (R Project for Statistical Computing). MROQC data were analyzed in SAS version 9.4 (SAS Institute Inc). A two‐sided *p* < .05 was considered significant. The 95% CIs for proportions were estimated using the Wilson score interval (prop.test). This study was exempted by the University of Michigan and VA Ann Arbor institutional review boards. The study followed the Strengthening the Reporting of Observational Studies in Epidemiology reporting guidelines for cohort studies.

## RESULTS

In the diagnosis‐based analysis, 546,343 SEER patients and 170,487 VA patients were diagnosed with localized PC and had sufficient information for risk‐group assignment. The proportion classified as low‐risk declined from 28.8% to 24.0% in SEER from 2010 to 2023 and from 32.3% to 21.9% in the VA from 2010 to 2024 (Figure 1 and Supporting Information S1: Table 1a,b). Among diagnosed low‐risk patients, receipt of neither radiation nor prostatectomy within 1 year increased from 33.6% to 83.4% in SEER and from 51.6% to 96.3% in the VA.

**FIGURE 1:**
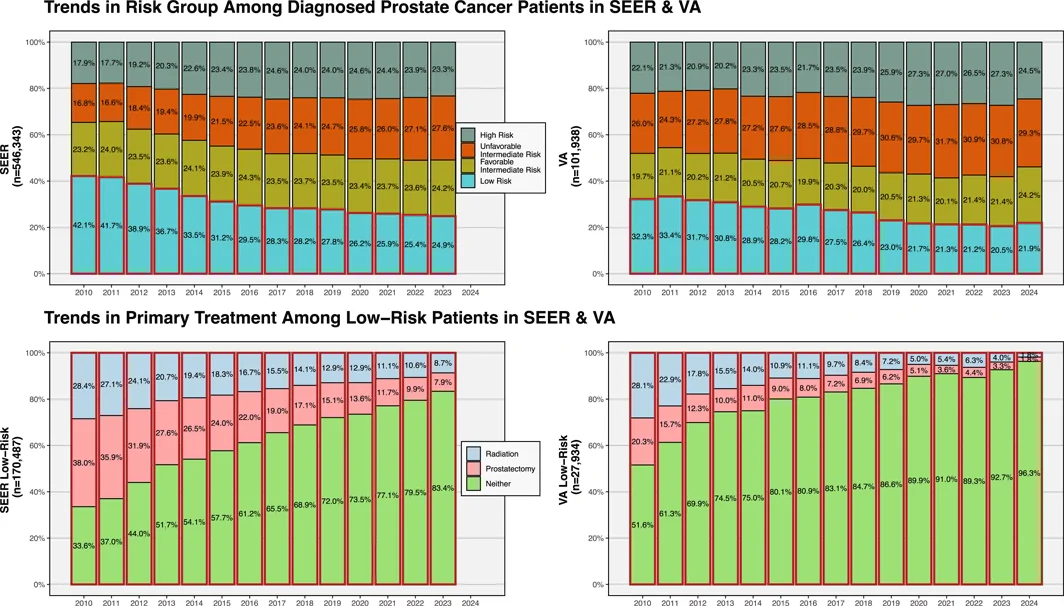
Diagnosis trends in risk group among 546,343 Surveillance, Epidemiology, and End Results Program (SEER) patients and 170,487 Veterans Affairs (VA) patients diagnosed with localized prostate cancer. Bottom row shows, among the low‐risk subset of these patients, primary treatment received within 1 year after diagnosis.

In the primary treatment analysis, a total of 207,196 radiation‐treated patients were then included: 170,213 in SEER treated from 2010 to 2023 (median [IQR] age, 69 [63–74] years); 34,635 in the VA treated from 2010 to 2024 (median [IQR] age, 68 [63–72]); and 2348 in MROQC treated from 2021 to 2025 (median [IQR] age, 71 [66–76] years). Cohort characteristics are summarized in Table 1.

**TABLE 1:** Cohort Characteristics.

Characteristic No. (%) of participants^a^	SEER (*n* = 170,213)	VA (*n* = 34,635)	MROQC (*n* = 2348)
Years evaluated	2010–2023	2010–2024	2021–2025
Age, median (IQR), y	69 (63–74)	68 (63–72)	71 (66–76)
Race			
Black	31,376 (18.4)	11,314 (32.6)	383 (16.3)
White	126,360 (74.2)	19,871 (57.4)	1844 (78.5)
Other^a^	9998 (5.9)	2165 (6.3)	48 (2.0)
Unknown	2479 (1.5)	1285 (3.7)	73 (3.1)
Pretreatment PSA, median (IQR), ng/mL	7.5 (5.4–11.7)	7.0 (5.0–11.0)	8 (5.9–12.5)
Pretreatment PSA, ng/mL			
<10	114,891 (67.5)	24,071 (69.5)	1492 (63.5)
10–20	37,307 (21.9)	7051 (20.4)	613 (26.1)
>20	18,015 (10.6)	3513 (10.1)	243 (10.3)
Clinical grade group			
1	36,177 (21.3)	5027 (14.5)	117 (5.0)
2	55,858 (32.8)	12,436 (35.9)	965 (41.1)
3	35,291 (20.7)	7809 (22.6)	751 (32.0)
4	24,648 (14.5)	5151 (14.9)	322 (13.7)
5	18,239 (10.7)	4212 (12.2)	193 (8.2)
Clinical T‐stage			
cT1‐T2a	146,553 (86.6)	29,065 (83.9)	2044 (87.1)
cT2b‐c	15,810 (9.3)	4421 (12.8)	195 (8.3)
cT3+	6836 (4.0)	1149 (3.3)	109 (4.6)
Risk group			
Low	28,129 (16.5)	3755 (10.8)	78 (3.3)
Favorable intermediate	37,920 (22.3)	6887 (19.9)	433 (18.4)
Unfavorable intermediate	50,305 (29.6)	12,639 (36.5)	1142 (48.6)
High	53,859 (31.6)	11,354 (32.8)	695 (29.6)
Treatment modality^b^			
EBRT	131,827 (77.4)	17,847 (84.4)	1504 (67.7)
Brachytherapy (± EBRT)	38,386 (22.6)	3289 (15.6)	718 (32.3)

*Note:* Races self‐reported throughout and in the other group include Alaska Native, American Indian, Middle Eastern, Asian, and Pacific Islander.

Abbreviations: EBRT, external beam radiation therapy; MROQC, Michigan Radiation Oncology Quality Consortium; PSA, prostate‐specifice antigen; SEER, Surveillance, Epidemiology, and End Results; VA, Veterans Administration.

^a^ Unless otherwise indicated.

^b^ 13,499 with unknown treatment modality in the VA cohort.

Among patients treated with radiation in SEER, the proportion with low‐risk PC dropped from 33.7% (95% CI, 32.9–34.5; 4481 of 13,295 patients) to 6.5% (95% CI, 6.1–6.9; 921 of 14,265 patients) from 2010 to 2023 (Figure 2 and Supporting Information S1: Table 2a). Adjusting for age and race, more recent treatment year was associated with a lower likelihood that a radiation‐treated patient had low‐risk disease (adjusted odds ratio [OR] per 5 years = 0.46; 95% CI, 0.45–0.47; *p* < .0001).

**FIGURE 2:**
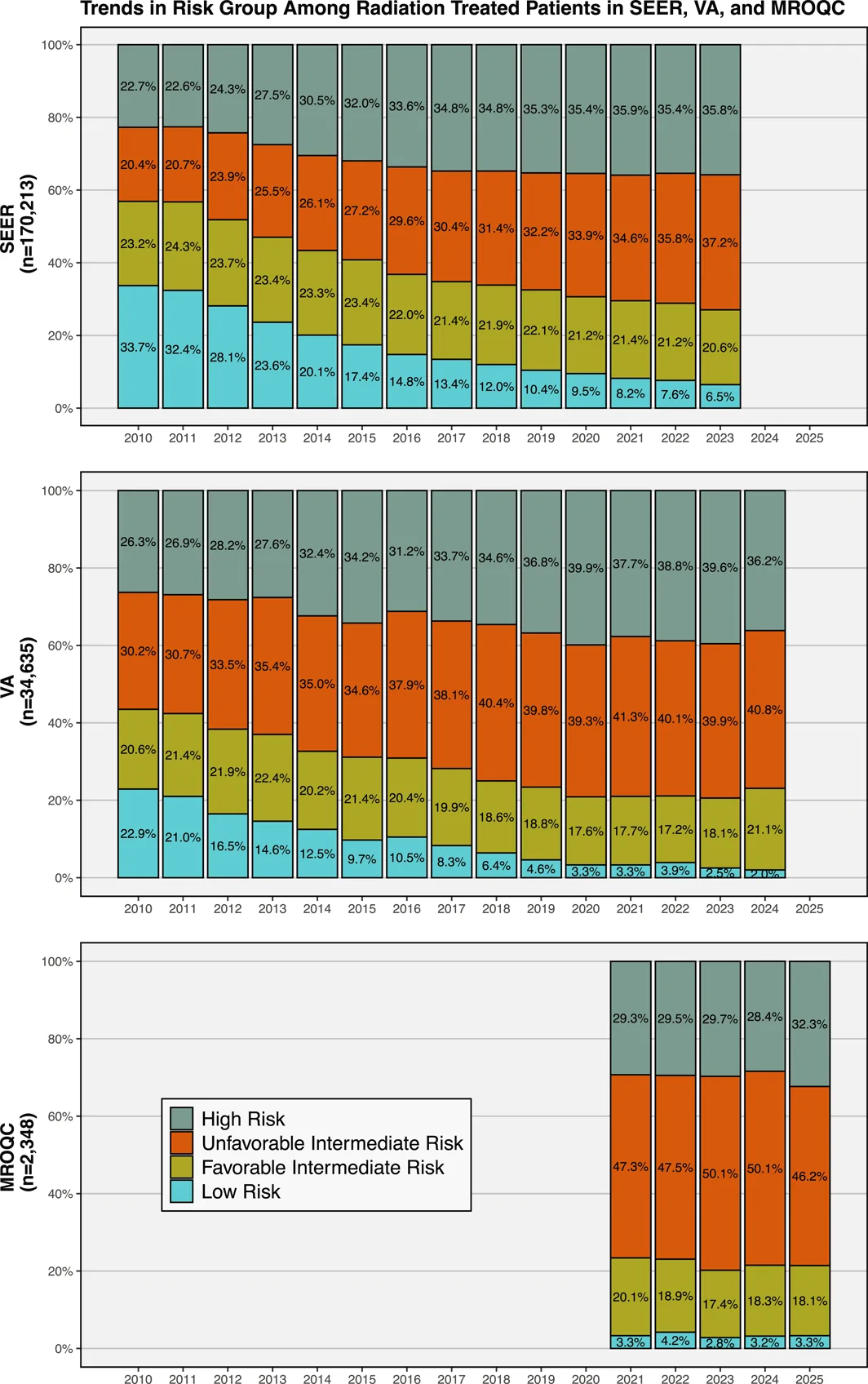
Trends in risk group among 170,213 Surveillance, Epidemiology, and End Results (SEER) patients, 34,635 Veterans Affairs (VA) patients, and 2348 Michigan Radiation Oncology Quality Consortium (MROQC) patients receiving radiation treatment for localized prostate cancer.

Within the VA, the percentage of patients receiving radiation for low‐risk PC dropped from 22.9% (95% CI, 21.5–24.4; 757 of 3301 patients) to 2.0% (95% CI, 0.4–3.5; 6 of 304) from 2010 to 2024 (Figure 2 and Supporting Information S1: Table 2b). As in SEER, more recent treatment year was associated with a lower likelihood that a radiation‐treated patient had low‐risk disease (OR per 5 years = 0.44; 95% CI, 0.41–0.46; *p* < .001).

Within MROQC, the percentage of patients receiving radiation for low‐risk PC remained low from 2021 to 2025, between a low of 2.8% in 2023 (95% CI, 1.7–4.6; 16 of 575) and a high of 4.2% in 2022 (95% CI, 2.6–6.6; 19 of 455) (Figure 2 and Supporting Information S1: Table 2c). In this narrower period, year of treatment was not associated with low‐risk disease among radiation‐treated patients (adjusted OR per 5 years = 0.97; 95% CI, 0.40–2.39; *p* = .95).

In all cohorts, older age was associated with a lower likelihood that a radiation‐treated patient had low‐risk PC while adjusting for treatment year and race (ORs per 5 years = 0.70, 0.70, and 0.77; 95% CI, 0.70–0.71, 0.68–0.72, and 0.68–0.89; all *p* < .001, in SEER, the VA, and MROQC, respectively). Black race was also associated with a lower likelihood that a radiation‐treated patient had low‐risk PC while adjusting for treatment year and age in SEER and the VA (ORs = 0.73 and 0.80; 95% CI, 0.70–0.75 and 0.74–0.88; *p* < .001 in SEER and the VA). Results of all multivariable analyses are shown in Supporting Information S1: Table 3.

In the overlapping years between cohorts: 2021–2023, the proportion of low‐risk cases was lower in both the VA (2.5%–3.9%) and MROQC (2.8%–4.2%) compared with SEER (6.5%‐8.2%; all *p* ≤ .01), with no significant differences between the VA and MROQC in the overlapping years (*p* ≥ .17).

Among 116 VA facilities, the proportion of radiation‐treated patients with low‐risk cancer at each facility was reported across three time periods: 2010–2014, 2015–2019, and 2020–2024 in Figure 3. Of these 116 facilities, only three treated a greater proportion of low‐risk prostate cancer in 2020 through 2024 compared with 2010 through 2014. Only five of 116 facilities had greater than 10% low‐risk PC among radiation‐treated patients in 2020 through 2024. In MROQC (2021–2025), only two of 26 facilities treated greater than 10% low‐risk PC from 2021 to 2025 (Supporting Information S1: Figure 1).

**FIGURE 3:**
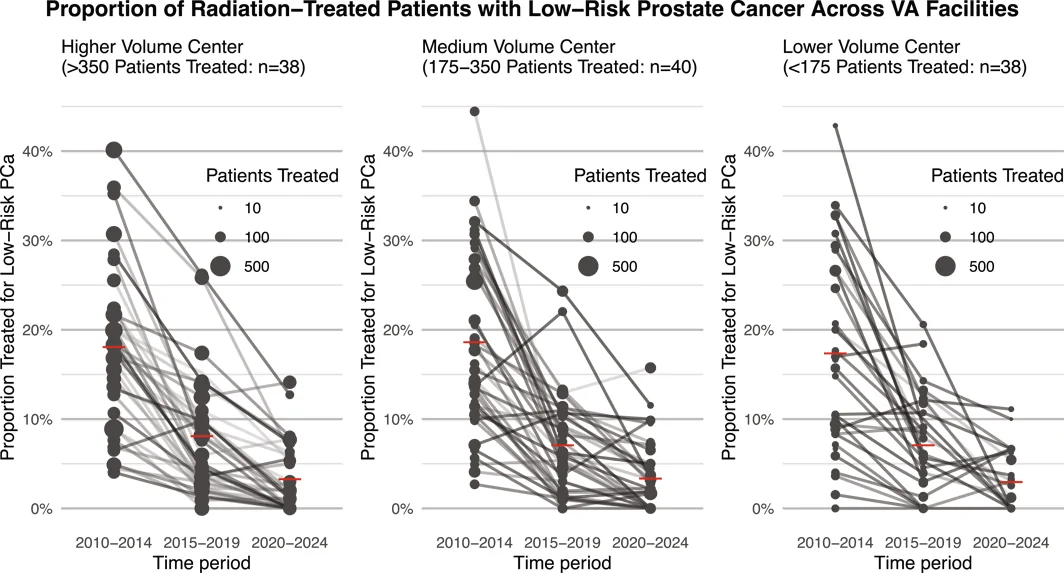
Slope graph showing the proportion of patients with radiation‐treated prostate cancer who had low‐risk disease within 116 Veterans Affairs (VA) facilities across three time periods: 2010–2014, 2015–2019, and 2020–2024. The y‐axis represents the proportion of all radiation‐treated patients with low‐risk prostate cancer. Facilities are grouped into tercile by total treatment volume from 2010–2024: higher (>350 patients), medium (175–350 patients), and lower (<175 patients), shown in separate panels. Each slope tracks a specific facility, bubble size reflects the number of patients treated in each time period, and red dash represents net proportion for that volume center. Facility‐period combinations with fewer than 10 patients were excluded.

## DISCUSSION

In this contemporary analysis of localized PC across population‐based, federal, and statewide cohorts in the United States, we found three complementary trends. First, among all patients diagnosed with localized PC in SEER and the VA, the proportion classified as low‐risk declined since 2010. Second, among diagnosed low‐risk patients, receipt of neither radiation nor prostatectomy within 1 year increased markedly. Third, among radiation‐treated patients, the proportion with low‐risk PC declined substantially across SEER, the VA, and MROQC. Together, these findings suggest a broad shift away from overtreatment of indolent prostate cancer.

Several factors likely influenced prostate cancer diagnosis across this period, including reduced PSA screening following the 2012 USPSTF recommendation, broader adoption of multiparametric magnetic resonance imaging and targeted biopsy, and adoption of the 2014 ISUP grade group system. However, the decline in low‐risk disease among radiation‐treated patients reflects more than diagnostic shifts alone. Although low‐risk PC became less common at diagnosis, the decline among radiation‐treated patients was substantially larger. In SEER, low‐risk disease among radiation‐treated patients dropped from 33.7% to 6.5%; in the VA, it dropped from 22.9% to 2.0%. Within MROQC, contemporary rates remained consistently low, below 5% from 2021 to 2025. These findings suggest that contemporary practice increasingly decouples early detection from unnecessary definitive treatment. It is also notable that in all cohorts the combination of unfavorable intermediate‐risk and high‐risk disease now makes up >70% of radiation‐treated patients.

Several treatment‐selection factors likely underlie the decline in radiation treatment for low‐risk disease. First, since 2010, major guidelines have increasingly endorsed active surveillance as the preferred strategy for low‐risk PC. Second, long‐term follow‐up from the PIVOT, PROTECT, and SPCG‐4 trials has reinforced the limited oncologic benefit of radical treatment in low‐risk patients.^12^, ^13^, ^14^ Third, the widespread availability and adoption of multiparametric prostate magnetic resonance imaging and targeted biopsy have improved discrimination of indolent and clinically significant disease, bolstering clinician confidence in deferring treatment.^15^ Finally, numerous quality improvement initiatives have helped disseminate active surveillance for low‐risk cancer throughout the country.^16^

These findings align with our prior work demonstrating reduced surgical overtreatment within SEER and MUSIC, suggesting a broad shift toward active surveillance across local treatment modalities.^4^ Together, these surgical and radiation treatment trends have important implications for the ongoing prostate cancer screening debate. Recommendations against routine screening have historically been driven by concerns regarding overtreatment‐related harms. Demonstrating that modern practice patterns substantially limit overtreatment despite ongoing diagnosis of low‐risk disease strengthens the argument that early detection can be pursued with less risk of unnecessary therapy.

Differences across cohorts may reflect variation in health system structure, patient populations, and quality improvement efforts. The particularly low rates of radiation treatment for low‐risk disease observed in the VA and MROQC compared with SEER suggest that integrated systems and statewide collaboratives may promote earlier adoption of guideline‐concordant practice and reductions in low‐value care.

Because Black men have been underrepresented in prostate cancer trials, the generalizability of practice changes remains an ongoing concern. In SEER and the VA, among radiation‐treated patients, Black race was associated with lower odds of having low‐risk PC after adjusting for age and treatment year, but treatment trends should be interpreted alongside potential differences in screening, stage and age at diagnosis, aggressiveness of disease, access to surveillance, and longitudinal care.

This analysis has several limitations. Relying exclusively on risk groups to define overtreatment is a narrow approach that overlooks life expectancy, competing health risks, and underlying tumor biology.^17^ As treatment of low‐risk cancer reaches very low levels nationally, sustained attention to residual overtreatment remains important, particularly in older or comorbid men for whom even higher‐risk disease may be unlikely to cause morbidity within their lifetime. Although our radiation‐treatment analysis adjusted for age, we did not examine the influence of age on risk‐group at diagnosis and how age at diagnosis has changed over time.

In addition, SEER clinical T‐stage was not reliably available for surgical patients in certain diagnosis years, requiring omission of T‐stage from SEER risk‐group assignment in the diagnosis‐based analysis. Finally, MROQC and the VA had fewer cases in the later years of the cohort, especially 2024 and 2025, and year‐to‐year differences in case ascertainment likely affects the stability of these estimates.

Our use of ORs, rather than risk ratios, was chosen to facilitate model convergence and to harmonize the analysis across the three registries. Because low‐risk disease was not a rare outcome in the earlier years of the study, the ORs reported here are larger in magnitude than the corresponding risk ratios and should not be interpreted as relative risks. Finally, although registry data carry inherent coding limitations, the parallel findings across three cohorts help mitigate these concerns and reinforce that practice patterns in localized PC have shifted substantially since 2010.

## CONCLUSIONS

Since 2010, low‐risk PC has become less common among localized PC diagnoses, and diagnosed low‐risk patients increasingly receive neither radiation nor prostatectomy within 1 year of diagnosis. Among patients receiving radiation, the proportion treated for low‐risk PC declined even more substantially across cohorts (SEER 33.7% to 6.5% by 2023; VA 22.9% to 2.0% by 2024) and remained below 5% in MROQC from 2021 to 2025. These trends parallel reductions in surgical overtreatment and reflect a broad shift in PC management away from treatment of indolent disease and toward active surveillance. Importantly, this marked reduction in unnecessary treatment informs the ongoing screening debate, as many concerns about PSA screening stem primarily from historical overtreatment rather than from early detection itself.

## AUTHOR CONTRIBUTIONS

**Steven M. Monda:** conceptualization; methodology; software; data curation; investigation; validation; formal analysis; supervision; visualization; writing – original draft; and writing – review & editing. **Varshini Odayar:** conceptualization and writing – review & editing. **Maggie Yin:** conceptualization; methodology; software; data curation; investigation; and formal analysis. **Rachel Tucker Gonzalez:** conceptualization; methodology; software; data curation; investigation; validation; and formal analysis. **Salvador Jaime–Casas:** conceptualization; writing – original draft; and writing – review & editing. **Karolyn M. Hopfensperger:** conceptualization; methodology; software; data curation; investigation; validation; formal analysis; supervision; and funding acquisition. **Danielle Kendrick:** conceptualization; methodology; data curation; and writing – review & editing. **Melissa A. Mietzel:** writing – review & editing; project administration; resources; conceptualization; and data curation. **Mazen Mislmani:** writing – review & editing and conceptualization. **Mark Zaki:** writing – review & editing and conceptualization. **Patrick W. McLaughlin:** writing – review & editing and conceptualization. **Todd M. Morgan:** writing – review & editing; conceptualization; and methodology. **William C. Jackson:** conceptualization; methodology; supervision; project administration; and writing – review & editing. **Matthew J. Schipper:** writing – review & editing; conceptualization; methodology; project administration; supervision; software; data curation; investigation; and formal analysis. **David K. Heimburger:** conceptualization; writing – review & editing; writing – original draft; and data curation. **Alex K. Bryant:** writing – review & editing; conceptualization; methodology; data curation; supervision; funding acquisition; and resources. **Lori J. Pierce:** writing – review & editing; project administration; supervision; investigation; and conceptualization. **Tudor Borza:** writing – review & editing; resources; project administration; funding acquisition; conceptualization; and methodology. **Robert T. Dess:** writing – review & editing; conceptualization; methodology; data curation; investigation; funding acquisition; project administration; and resources.

## CONFLICT OF INTEREST STATEMENT

Melissa Mietzel: other relationship with Blue Cross Blue Shield of Michigan. Todd Morgan: consulting or advisory role with Foundation Medicine, Merck, Pfizer, Johnson & Johnson/Janssen, and Envision Sciences. William Jackson: research funding from Janssen. Matthew Schipper: consulting/advisory for Innovative Analytics. David Heimburger: Lilly, Novartis, Boston Scientific Intuitive Surgical, Johnson & Johnson/Janssen McKesson, ABBVIE/ABBOTT Regeneron. Lori Pierce: patents, royalties, other intellectual property with UpToDate, PFS Genomics; uncompensated relationships: Bristol‐Myers Squibb, BCRF Scientific Advisory Board, and Damon Runyan Cancer Research Foundation. Tudor Borza: research funding from AHRQ.

## Supporting information

Supporting Information S1

## Data Availability

The data that support the findings of this study are available on request from the corresponding author. The data are not publicly available due to privacy or ethical restrictions.
