# Supplementary material for: Population‐level diagnostic and radiation treatment trends of low‐risk prostate cancer
Source: Cancer. 2026 Oct 26;132(21):e70603. doi: 10.1002/cncr.70603 (PMC13696980; doi:10.1002/cncr.70603)
Supplement: Supplementary file 1 [file CNCR-132-e70603-s001.docx]

**Supplementary Figure 1.** Bubble plot representing 26 facilities within MROQC providing radiation treatment. Bubble size represents the number of radiation treatments for localized prostate cancer provided and vertical location represents the proportion radiation-treated patients with low-risk prostate cancer.

**Supplementary Tables 1 a and b.** Clinical risk group at diagnosis among patients with localized prostate cancer diagnosed between a) 2010 and 2023 in SEER and b) 2010 and 2024 the VA. Data represented in Figure 1.

a) SEER: Localized prostate cancer diagnoses

| **Clinical Risk** | **2010** | **2011** | **2012** | **2013** | **2014** | **2015** | **2016** | **2017** | **2018** | **2019** | **2020** | **2021** | **2022** | **2023** |
| --- | --- | --- | --- | --- | --- | --- | --- | --- | --- | --- | --- | --- | --- | --- |
| Low | 11825 (28.8%) | 12243 (29.3%) | 9926 (28.2%) | 9579 (28.0%) | 8068 (25.8%) | 8244 (24.5%) | 9686 (27.8%) | 9999 (26.7%) | 10730 (26.7%) | 11348 (26.2%) | 9330 (25.0%) | 11266 (24.8%) | 11143 (24.4%) | 10831 (24.0%) |
| Favorable Intermediate | 10398 (25.3%) | 10563 (25.3%) | 8189 (23.3%) | 7564 (22.1%) | 6653 (21.3%) | 6762 (20.1%) | 7458 (21.4%) | 7774 (20.8%) | 8392 (20.9%) | 8980 (20.7%) | 7729 (20.7%) | 9669 (21.3%) | 9631 (21.1%) | 9810 (21.7%) |
| Unfavorable Intermediate | 8925 (21.7%) | 9001 (21.5%) | 8098 (23.0%) | 8053 (23.5%) | 7434 (23.8%) | 8298 (24.7%) | 6627 (19.0%) | 7547 (20.2%) | 8487 (21.1%) | 9164 (21.2%) | 8239 (22.1%) | 10097 (22.2%) | 10643 (23.3%) | 10758 (23.8%) |
| High | 9925 (24.2%) | 10000 (23.9%) | 8932 (25.4%) | 9074 (26.5%) | 9109 (29.1%) | 10277 (30.6%) | 11094 (31.8%) | 12115 (32.4%) | 12604 (31.3%) | 13792 (31.9%) | 12009 (32.2%) | 14364 (31.6%) | 14177 (31.1%) | 13710 (30.4%) |

b) VA: Localized prostate cancer diagnoses

| **Clinical Risk** | **2010** | **2011** | **2012** | **2013** | **2014** | **2015** | **2016** | **2017** | **2018** | **2019** | **2020** | **2021** | **2022** | **2023** | **2024** |
| --- | --- | --- | --- | --- | --- | --- | --- | --- | --- | --- | --- | --- | --- | --- | --- |
| **Low** | 2695 (32.3%) | 2922 (33.4%) | 2473 (31.7%) | 2239 (30.8%) | 1908 (28.9%) | 2028 (28.2%) | 2320 (29.8%) | 2293 (27.5%) | 2280 (26.4%) | 1855 (23.0%) | 1215 (21.7%) | 1221 (21.3%) | 1258 (21.2%) | 902 (20.5%) | 325 (21.9%) |
| **Favorable Intermediate** | 1645 (19.7%) | 1845 (21.1%) | 1573 (20.2%) | 1540 (21.2%) | 1355 (20.5%) | 1490 (20.7%) | 1550 (19.9%) | 1692 (20.3%) | 1727 (20.0%) | 1655 (20.5%) | 1190 (21.3%) | 1154 (20.1%) | 1268 (21.4%) | 939 (21.4%) | 359 (24.2%) |
| **Unfavorable Intermediate** | 2170 (26.0%) | 2131 (24.3%) | 2121 (27.2%) | 2017 (27.8%) | 1797 (27.2%) | 1989 (27.6%) | 2219 (28.5%) | 2399 (28.8%) | 2568 (29.7%) | 2465 (30.6%) | 1660 (29.7%) | 1817 (31.7%) | 1830 (30.9%) | 1354 (30.8%) | 435 (29.3%) |
| **High** | 1845 (22.1%) | 1862 (21.3%) | 1628 (20.9%) | 1468 (20.2%) | 1541 (23.3%) | 1695 (23.5%) | 1692 (21.7%) | 1958 (23.5%) | 2063 (23.9%) | 2085 (25.9%) | 1527 (27.3%) | 1548 (27.0%) | 1574 (26.5%) | 1200 (27.3%) | 364 (24.5%) |

**Supplementary Tables 2 a, b, and c.** a) Clinical risk group among patients receiving radiation in SEER for each year between 2010 and 2023, b) in the VA between 2010 to 2024, and c) in MROQC between 2021 and 2025. Data represented in Figure 2.

a) **SEER: Localized prostate cancer treatment with radiation**

| **Clinical Risk** | **2010** | **2011** | **2012** | **2013** | **2014** | **2015** | **2016** | **2017** | **2018** | **2019** | **2020** | **2021** | **2022** | **2023** |
| --- | --- | --- | --- | --- | --- | --- | --- | --- | --- | --- | --- | --- | --- | --- |
| Low | 4481 (33.7%) | 4318 (32.4%) | 3002 (28.1%) | 2376 (23.6%) | 1864 (20.1%) | 1758 (17.4%) | 1538 (14.8%) | 1493 (13.4%) | 1455 (12.0%) | 1412 (10.4%) | 1150 (9.5%) | 1212 (8.2%) | 1149 (7.6%) | 921 (6.5%) |
| Favorable Intermediate | 3082 (23.2%) | 3245 (24.3%) | 2529 (23.7%) | 2353 (23.4%) | 2161 (23.3%) | 2362 (23.4%) | 2297 (22.0%) | 2375 (21.4%) | 2654 (21.9%) | 2999 (22.1%) | 2565 (21.2%) | 3164 (21.4%) | 3196 (21.2%) | 2938 (20.6%) |
| Unfavorable Intermediate | 2712 (20.4%) | 2755 (20.7%) | 2550 (23.9%) | 2563 (25.5%) | 2426 (26.1%) | 2748 (27.2%) | 3082 (29.6%) | 3381 (30.4%) | 3809 (31.4%) | 4363 (32.2%) | 4113 (33.9%) | 5118 (34.6%) | 5385 (35.8%) | 5300 (37.2%) |
| High | 3020 (22.7%) | 3014 (22.6%) | 2589 (24.3%) | 2765 (27.5%) | 2828 (30.5%) | 3227 (32.0%) | 3505 (33.6%) | 3865 (34.8%) | 4220 (34.8%) | 4784 (35.3%) | 4295 (35.4%) | 5315 (35.9%) | 5326 (35.4%) | 5106 (35.8%) |

b) VA: Localized prostate cancer treatment with radiation

| **Clinical Risk** | **2010** | **2011** | **2012** | **2013** | **2014** | **2015** | **2016** | **2017** | **2018** | **2019** | **2020** | **2021** | **2022** | **2023** | **2024** |
| --- | --- | --- | --- | --- | --- | --- | --- | --- | --- | --- | --- | --- | --- | --- | --- |
| **Low** | 757 (22.9%) | 670 (21.0%) | 440 (16.5%) | 346 (14.6%) | 268 (12.5%) | 221 (9.7%) | 258 (10.5%) | 222 (8.3%) | 191 (6.4%) | 134 (4.6%) | 61 (3.3%) | 66 (3.3%) | 79 (3.9%) | 36 (2.5%) | 6 (2.0%) |
| **Favorable Intermediate** | 678 (20.6%) | 680 (21.4%) | 585 (21.9%) | 532 (22.4%) | 431 (20.2%) | 488 (21.4%) | 504 (20.4%) | 535 (19.9%) | 556 (18.6%) | 542 (18.8%) | 328 (17.6%) | 351 (17.7%) | 349 (17.2%) | 264 (18.1%) | 64 (21.1%) |
| **Unfavorable Intermediate** | 997 (30.2%) | 978 (30.7%) | 896 (33.5%) | 840 (35.4%) | 748 (35.0%) | 789 (34.6%) | 936 (37.9%) | 1,026 (38.1%) | 1,206 (40.4%) | 1148 (39.8%) | 734 (39.3%) | 820 (41.3%) | 814 (40.1%) | 583 (39.9%) | 124 (40.8%) |
| **High** | 868 (26.3%) | 857 (26.9%) | 754 (28.2%) | 655 (27.6%) | 692 (32.4%) | 780 (34.2%) | 771 (31.2%) | 908 (33.7%) | 1,035 (34.6%) | 1,064 (36.8%) | 746 (39.9%) | 748 (37.7%) | 786 (38.8%) | 580 (39.6%) | 110 (36.2%) |

c) MROQC: Localized prostate cancer treatment with radiation

| **Clinical Risk** | **2021** | **2022** | **2023** | **2024** | **2025** |
| --- | --- | --- | --- | --- | --- |
| **Low** | 11  (3.3%) | 19  (4.2%) | 16  (2.8%) | 21  (3.2%) | 11  (3.3%) |
| **Favorable Intermediate** | 68  (20.1%) | 86  (18.9%) | 100  (17.4%) | 119  (18.3%) | 60  (18.1%) |
| **Unfavorable Intermediate** | 160  (47.3%) | 216  (47.5%) | 288  (50.1%) | 325  (50.1%) | 153  (46.2%) |
| **High** | 99  (29.3%) | 134 (29.5%) | 171 (29.7%) | 184 (28.4%) | 107 (32.3%) |

**Supplementary Table 3.** Multivariable analysis modeling the likelihood that a radiation-treated patient had low-risk prostate cancer among all patients treated with radiation for localized prostate cancer. Low-risk status (low-risk vs. non-low-risk) was modeled with logistic regression, with treatment year (per 5 years), age (per 5 years), and race as fixed effects. A random intercept was included for SEER-17 registry in the SEER model and for treating facility in the VA and MROQC models.

| Variable | SEER 2010-2023  (n=170,213) | | VA 2010-2024  (n=34,635) | | MROQC 2021-2025  (n=2,348) | |
| --- | --- | --- | --- | --- | --- | --- |
|  | OR (95% CI) | P value | OR (95% CI) | P value | OR (95% CI) | P value |
| Year (+5 y) | 0.46 (0.45-0.47) | <0.001 | 0.44 (0.41-0.46) | <0.0001 | 0.97 (0.40-2.39) | 0.95 |
| Age (+5 y) | 0.70 (0.69-0.71) | <0.001 | 0.70 (0.68-0.72) | <0.001 | 0.77 (0.68-0.89) | <0.001 |
| Race |  |  |  |  |  |  |
| White | 1 [Reference] | NA | 1 [Reference] | NA | 1 [Reference] | NA |
| Black | 0.73 (0.70-0.75) | <0.001 | 0.80 (0.74-0.88) | <0.001 | 0.94  (0.49-1.67) | 0.62 |
| Other | 0.82 (0.77-0.88) | <0.001 | 1.02 (0.86-1.21) | 0.8 | 0.91  (0.10-3.49) | 0.76 |
| Unknown/Missing | 1.27 (1.14-1.42) | <0.001 | 1.03 (0.85-1.25) | 0.8 | 1.72  (0.55-4.20) | 0.30 |
